# Contact rate modulates foraging efficiency in leaf cutting ants

**DOI:** 10.1038/srep18650

**Published:** 2015-12-21

**Authors:** S. Bouchebti, S. Ferrere, K. Vittori, G. Latil, A. Dussutour, V. Fourcassié

**Affiliations:** 1 Université Fédérale de Toulouse Midi-Pyrénées, UPS Centre de Recherches sur la Cognition Animale–UMR 5169, 118 route de Narbonne, F-31062 Toulouse cedex 9, France; 2 CNRS Centre de Recherches sur la Cognition Animale – UMR 5169, 118 route de Narbonne, F-31062 Toulouse cedex 9, France

## Abstract

Lane segregation is rarely observed in animals that move in bidirectional flows. Consequently, these animals generally experience a high rate of head-on collisions during their journeys. Although these collisions have a cost (each collision induces a delay resulting in a decrease of individual speed), they could also have a benefit by promoting information transfer between individuals. Here we explore the impact of head-on collisions in leaf-cutting ants moving on foraging trails by artificially decreasing the rate of head-on collisions between individuals. We show that head-on collisions do not influence the rate of recruitment in these ants but do influence foraging efficiency, i.e. the proportion of ants returning to the nest with a leaf fragment. Surprisingly, both unladen and laden ants returning to the nest participate in the modulation of foraging efficiency: foraging efficiency decreases when the rate of contacts with both nestbound laden or unladen ants decreases. These results suggest that outgoing ants are able to collect information from inbound ants even when these latter do not carry any leaf fragment and that this information can influence their foraging decisions when reaching the end of the trail.

Although fluid-like collective movement are observed in many organisms, only humans and social insects are known to move in bidirectional flows^1^ and thus to potentially experience a high rate of collisions during their movement. To avoid these collisions and move more smoothly, humans^2^ and some species of ants^3^ and open-air foraging termites^4^,^5^ spatially segregate their flows and travel in unidirectional lanes. Most species of ants and termites however do not show lane segregation and this implies a certain cost for their colony. Indeed, each collision induces a short delay in the progression of individuals and thus significantly decreases their speed, which in turn, when summed over the thousands of collisions that occur along a foraging trail, eventually decreases the rate of food return to the nest^6^. These collisions however can also promote information transfer between individuals and thus benefit their colonies. Hence, in the black garden ant *Lasius niger* repeated contacts with nestmates or with glass beads coated with their cuticular hydrocarbons reinforce path choice^7^ and reduce the frequency of trail pheromone deposits by ants, thus acting as a negative feedback allowing the avoidance of traffic congestion on foraging trails^8^,^9^. In addition to the contacts occurring on foraging trails, the contacts occurring inside the nest galleries prolonging foraging trails can also be important in conveying information about the type of food available outside the nest^10^ or in regulating the recruitment to the food sources being exploited. For example, in the seed harvester ant *Pogonomyrmex barbatus* the departure rate of foragers from the nest depends on the rate of encounters with successful foragers coming back to the nest^11^,^12^ or on the rate at which glass beads coated with a combination of worker cuticular hydrocarbon and seed odour are introduced inside the nest galleries^13^.

Leaf-cutting ants (LCA) are a good example of ant species in which there is very little segregation between ants moving in opposite directions on foraging trails. The flows are partly intermingled, which results in a high rate of head-on collisions^14^. The role of contacts occurring during these collisions however is equivocal. On the one hand, Burd^15^ found no impact on recruitment in the leaf cutting ant *Atta colombica* when he removed during two hours all laden ants travelling on a foraging trail. On the other hand Dussutour *et al.*^16^ observed a significant increase in foraging efficiency, i.e. in the proportion of ants returning to the nest with a leaf fragment, when they increased the rate of contacts between workers moving in opposite directions by decreasing the width of their foraging trail. These two studies suggest therefore that in LCA contact rates have no impact on recruitment but may have one on resource collection. That contacts can inform outbound workers on the type of resource being exploited has indeed been shown by Farji-Brener *et al.*^17^ who provided evidence that the probability for an outgoing ant finding and collecting a new resource is higher if it has previously contacted an inbound ant carrying this resource. The fact that outgoing ants tend to contact more workers at the beginning of their foraging activity suggests indeed that they are seeking through this means information about the resource being exploited^18^. However we do not know yet if the *rate* of head-on contacts, i.e. the number of contacts experienced per unit time, whether with laden or unladen nestbound workers, can modulate the motivation of outbound foraging ants to collect and carry a load, independent of the density of trail pheromone deposits on the trail. In fact, in Dussutour *et al*’s experiment the increase in foraging efficiency on a narrow bridge could be equally explained by an increase in the rate of contacts between workers and/or by an increase in the amount of trail pheromone which is known to have a stimulating effect on foraging ants. Here we explore in LCA the impact of a change in the rate of head-on collisions with either laden or unladen returning workers, independent of the amount of trail pheromone, both on the modulation of recruitment and on foraging efficiency. We examine the effect of a decrease in the rate of head-on contacts or of a total absence of these contacts, either by manually removing laden or unladen inbound ants from the trail (experiment 1) or by forcing them to move in two segregated lanes (experiment 2).

## Results

All experiments were performed on two *Atta laegivata* colonies that were maintained in the lab in constant conditions.

### Experiment 1

In this experiment, a food source was connected to a colony of *Atta laegivata* by a bridge. Ant traffic was recorded on the bridge during two phases of 20 minutes. In the first phase no manipulation occurred (control phase) while in the second phase (experimental phase), also lasting 20 minutes, either one unladen ant out of two (DUnl Treatment = Decrease Unladen ants), or one laden ant out of two (DL Treatment = Decrease Laden ants) or no ants (CONT Treatment = CONTrol) were removed.

First, we ensured that we were able to manipulate the number of contacts between outbound ants and laden and unladen inbound ants during the experimental phase.

The proportion of contacts between outbound ants and laden and unladen inbound ants differed significantly among treatments (χ^2^ = 27.994, *P* < 0.001). As expected, the proportion of contacts with laden ants was higher in the DUnl Treatment (15.10%) and lower in the DL Treatment (6.08%) than in the CONT Treatment (11.12%). In the CONT treatment, an outgoing ant passed on a 15cm trail section on average 7.09 ± 4.81 (mean ± SD) unladen ants and 0.96 ± 1.28 laden ants and contacted 4.155 ± 2.635 unladen ants and 0.520 ± 0.795 laden ants. The proportion of laden/unladen ants contacted did not differ significantly from the proportion of laden/unladen ants in the inbound flow for the three treatments (χ^2^ = 0.343, *P* = 0.558; χ^2^ = 0.011, *P* = 0.918; χ^2^ = 0.795, *P* = 0.373; for the CONT, DL and DUnl treatment respectively), which means that outbound ants did not preferentially attempt to contact either category of ants.

Overall, there was a decrease in the number of ants exiting the nest between the control phase and the experimental phase of the experiment and this decrease did not differ between treatments (Table 1: GLMM, treatment effect: *F*_2,56_ = 1.412, *P* = 0.252). As for the change in foraging efficiency between the two phases of the experiment the GLMM shows that it was significantly different between the three treatments (Table 1: GLMM, treatment effect: *F*_2, 56_ = 7.469, *P* = 0.001). The difference in foraging efficiency was significantly higher in the CONT treatment than in the DL (Tukey post-hoc: *z* = −2.436, *P* = 0.044) and DUnl treatment (Tukey post-hoc: *z* = −3.817, *P* < 0.001) but not significantly different between the DL and DUnl treatment (Tukey post-hoc: *z* = −1.380*, P* = 0.502).

### Experiment 2

In order to study foraging efficiency without any contacts on the foraging trail, we isolated outbound and inbound traffic by using two bridges that linked the colony to the food source. There was therefore a total absence of contacts between outbound and inbound ants.

In these conditions we found that foraging efficiency was significantly lower than in the control phase of experiment 1 (all treatments pooled) in which the rate of contacts between ants was not manipulated (GLMM, treatment effect: *F*_1, 77_ = 22.242, *P* < 0.001).

## Discussion

The results of our two experiments show that the rate of contacts between ants moving in opposite directions had no impact on recruitment in leaf cutting ants, as already shown by Burd^15^, but had a strong one on foraging efficiency. The difference in foraging efficiency between the two phases of the experiment was significantly higher in the control treatment than in the two treatments in which the rate of head-on contacts was reduced. Foraging efficiency increased in the control treatment, as it usually does over time^18^, but there was only a small difference between the two phases of the experiment in the two experimental treatments. This suggests that the decision to cut or collect and transport a leaf fragment for an ant arriving at the end of a trail depends on the rate of contacts it has experienced when travelling on this trail.

LCA use mass recruitment through trail pheromone to recruit nestmates to the plants they exploit^19^. Experiments with artificial trails show that the rate of recruitment, i.e. the change in the number of ants exiting the nest over time, is directly linked to the increase in the amount of pheromone deposited by ants^20^. Trail pheromone should thus be the main signal regulating recruitment in LCA and the fact that recruitment was not influenced by the rate of contacts in our experiments should come as no surprise. On the other hand, trail pheromone does not seem to be involved in the variation of foraging efficiency observed between treatments. In fact, since the trail pheromone of LCA has a low evaporation rate^21^, the removal of ants during the experimental phase in the two experimental treatments, 50min after the initiation of recruitment, should have little or no impact on the amount of pheromone on the trail. That it was indeed the case, is shown by the fact that the number of ants exiting the nest in the experimental phase of the experiment, which mainly depends on the amount of pheromone on the trail (see above), was not significantly different between treatments (*F*_2,56_ = 1.09, *P* = 0.343).

Interestingly, both contacts with laden and unladen ants had an influence on foraging efficiency, suggesting that in LCA information transfer occurs during head-on contacts, whether or not inbound ants carry a leaf fragment. Unladen ants returning to the nest may have their mandibles smeared with plant sap, either because they directly ingested plant juice from the surface of cut leaves^22^ or because they took part in the cutting of the vegetation without subsequently transporting the fragment they cut^23^. This contrasts with the situation of harvester ants in which only laden workers have an impact on recruitment^11^. The difference between the two species may be explained by the fact that harvester ants do not cut the seeds they transport. Unladen workers of harvester ants are thus unlikely to convey chemical information about the resource being exploited. Our result provides some support to the hypothesis proposed by Roces^24^ and Bollazzi & Roces^18^ to explain the higher proportion of unladen ants in the initial phase of recruitment in LCA. If, as shown in our experiment, unladen ants are as much able as laden ants to convey information to outbound ants about the resource being exploited, they could indeed accelerate information transfer at the beginning of recruitment since they move faster than laden ants. Nevertheless, our results suggest that a contact with a laden ant seems to have a higher impact on foraging efficiency than a contact with an unladen ant. In fact, although in the DL treatment removing one laden ant out of two corresponded to removing only 6.50% of the ants from the inbound flow, in the DUnl treatment removing one unladen ant out of two corresponded to removing 43.5% of the ants from the inbound flow. In spite of this huge difference however, the change in foraging efficiency between the control phase and the experimental phase of the experiment was not significantly different between the two treatments. Direct contacts of outbound ants with the leaf fragments carried by inbound ants probably conveyed more information on the type of substrate harvested than mere contacts with inbound ants’ mandibles or the perception of the substrate odour^25^.

Experiment 2 shows that in LCA, contrary to what is observed in harvester ants^13^, the contacts on the foraging trail but not inside the nest are important to regulate foraging efficiency. In fact, contrary to what happened in experiment 1, the inbound flow was not manipulated in experiment 2 and ants departing from the foraging area were not impeded on return to the nest. Therefore, the rate of contacts between returning ants and their nestmates inside the nest should not vary compared to the control situation in which the flow was bidirectional. The decrease in foraging efficiency observed in this experiment can thus only be explained by the absence of head-on contacts on the trail due to the segregation between the outbound and inbound flows.

LCA use a dendritic network of stable physical trails as a basis to exploit the resources located in the environment surrounding their nest^26^. A trail departing from the nest can therefore bifurcate several times and lead to different types of resources that can be exploited simultaneously^19^. It thus makes sense that the modulation of foraging efficiency, i.e. of the probability of an ant to collect a leaf fragment at the end of a trail, should occur directly on the trails, through head-on contacts, rather than inside the nest. Ants exiting the nest could thus use both the rate of contacts with inbound ants and the odour and taste of the leaf fragments transported by their nestmates^25^,^27^ as criteria to decide whether to collect a resource at the end of a trail.

## Methods

Two mature colonies of *Atta laevigata* were used in the experiments. The colonies were maintained at 30 ± 10% RH and 25 ± 2 °C under a 12:12 L:D cycle. They were provided with water and fed *ad libitum* with fresh leaves of scarlet firethorn. The fungus of each colony was kept in two clear PVC boxes (W × L × H: 27 × 24 × 27cm) that were both connected to an open PVC box (W × L × H: 21 × 47 × 20cm) that was used as a foraging area. The walls of the boxes were coated with Fluon® to prevent the ants from escaping.

### Experiment 1

One hour before the beginning of a test, all vegetation remaining in the foraging area was removed. Then, a PVC bridge (L × W: 70 × 1.5cm) was placed between the foraging area and a second PVC box (W × L × H: 30 × 46 × 12cm) in which 9g of brambles, pre-cut in 10mm diameter disks, were placed. Thirty minutes after placing the bridge, a video camera (JVC HDD everio) placed centrally over the bridge started to record the flow of ants during 40min. The experiment was divided in two different phases. During the first 20min no manipulation was performed (control phase), whereas during the last 20min (experimental phase) one of the three following treatments was applied. In the first treatment (CONT = CONTrol) the experimental phase was identical to the control phase, while in the second treatment (DL = Decrease Laden ants) and in the third treatment (DUnl = Decrease Unladen ants) one laden ant out of two and one unladen ant out of two respectively, was gently removed from the flow of returning ants with forceps, as soon as they climbed on the bridge leading to the nest. This method has been used in previous studies^15^,^16^,^28^ and in our experiment it did not appear to elicit alarm behaviour^29^ and disturb ant activity: ants continued to walk normally on the bridge. Ten replicates were performed for each treatment and each colony. Note that there was always some brambles left in the foraging area at the end of each replicate of the experiment.

To check for the effect of ant removal on the rate of contacts between ants moving in opposite directions, we followed a sample of 10 outbound ants crossing a 15 cm section in the centre of the bridge during the experimental phase of each replicate for each treatment (N = 600 ants in total) and counted the number of laden and unladen ants returning to the nest that were passed by each ant followed as well as the number of head-on contacts they made.

### Experiment 2

To test for the effect of the total absence of contacts between outbound and inbound ants, a second experiment was performed in which ants were forced to move in two segregated lanes. Two PVC bridges of the same length and width as in experiment 1 were placed side by side between the colony foraging area next to the nest and the box containing the substrate. One bridge had its pillar inside the box containing the substrate coated with Fluon® (outbound flow), while the same was done for the opposite pillar of the other bridge, the one inside the colony foraging area (nestbound flow). Therefore, ants exiting the nest were forced to walk on one bridge to have access to the leaves and those returning to the nest were forced to walk on the other bridge^30^. Ideally we should have had a control phase with a bidirectional traffic and an experimental phase with a unidirectional traffic. Changing the setup between the two phases of the experiment however would have seriously disturbed the ants and would have precluded the comparison with experiment 1. We thus followed the same experimental protocol as in experiment 1 but always with two segregated flows. We placed 9g of brambles in the foraging area and ants had access to this latter first during a period of 30 minutes (familiarization phase) and then during a period of 40min, corresponding to the 20min of the control phase and the 20min of the experimental phase of experiment 1. Ten replicates of the experiment were performed for each colony.

### Data collected

The number of laden and unladen ants travelling in both directions was counted during the two phases of the two experiments. The number of outbound ants was used to assess recruitment. The number of inbound ants was then corrected in the two treatments DL and DUnl of experiment 1 by adding the ants removed at the departure from the foraging area and foraging efficiency was measured as the ratio of inbound laden ants on the total number of inbound ants. Since a high variation of activity was sometimes observed between replicates, we used the difference in the value of the response variables between the experimental phase and the control phase to examine the effect of each treatment. This difference, expressed in percentage for the number of outbound ants and in absolute value for foraging efficiency, was then compared between treatments.

### Statistical analyses

We used a GLMM to compare the response variables between treatments. Colony was entered as a random factor and a Tukey *post hoc* test was used for pairwise comparisons between treatments. The proportion of contacts between outbound ants and laden and unladen ants was compared between the three treatments of experiment 1 by a χ^2^ test for independence. We also compared with a χ^2^ test the proportion of laden/unladen ants contacted to the proportion of laden/unladen ants that were passed. This allowed us to test whether outbound ants preferentially contacted inbound laden or unladen ants in the three treatments of experiment 1. To assess the effect of the total absence of contacts between ants moving in opposite direction, the foraging efficiency observed in the experimental phase of experiment 2 (i.e. without contacts) was compared with that observed in the control phase of experiment 1 (all three treatments pooled) with a GLMM. All statistical tests were run with the software R 2.13.1^31^.

## Additional Information

**How to cite this article**: Bouchebti, S. *et al.* Contact rate modulates foraging efficiency in leaf cutting ants. *Sci. Rep.* **5**, 18650; doi: 10.1038/srep18650 (2015).

## Figures and Tables

**Table 1:** Effect of treatment on the percentage difference in the number of ants exiting the nest and in foraging efficiency between the experimental and control phase of each experiment.

	CONT^*1*^	DL^*1*^	DUnl^*1*^	*GLMM*^*2*^
% difference in outbound ants^3^	−13.317 ± 4.645^a^	−9.944 ± 4.587^a^	−15.670 ± 6.480^a^	*NS*
Foraging efficiency^*3*^	0.046 ± 0.021^a^	0.015 ± 0.019^b^	−0.002 ± 0.014^b^	*0.001*

^1^mean  ±  CI_*0.95.*_

^2^treatment effect (GLMM with treatment entered as fixed effect and colony entered as random effect).

^3^means sharing the same letter are not significantly different (*post-hoc* Tukey test, *P* < 0.05).

CONT = control; DL = decrease laden ants; DUnL = decrease unladen ants; *N* = 20 replicates per treatment.
